# Development of a landscape integrity model framework to support regional conservation planning

**DOI:** 10.1371/journal.pone.0195115

**Published:** 2018-04-03

**Authors:** Leroy J. Walston, Heidi M. Hartmann

**Affiliations:** Environmental Science Division, Argonne National Laboratory, Lemont, Illinois, United States of America; Michigan State University, UNITED STATES

## Abstract

Land managers increasingly rely upon landscape assessments to understand the status of natural resources and identify conservation priorities. Many of these landscape planning efforts rely on geospatial models that characterize the ecological integrity of the landscape. These general models utilize measures of habitat disturbance and human activity to map indices of ecological integrity. We built upon these modeling frameworks by developing a Landscape Integrity Index (*LII*) model using geospatial datasets of the human footprint, as well as incorporation of other indicators of ecological integrity such as biodiversity and vegetation departure. Our *LII* model serves as a general indicator of ecological integrity in a regional context of human activity, biodiversity, and change in habitat composition. We also discuss the application of the *LII* framework in two related coarse-filter landscape conservation approaches to expand the size and connectedness of protected areas as regional mitigation for anticipated land-use changes.

## Introduction

Recently there is increasing emphasis by landscape ecologists and land managers on incorporating broad-scale ecosystem health information into management decisions that cross land ownership boundaries. Several U.S. agencies are partnering with local governments, non-profit, and other groups in using a landscape approach for identifying important areas for restoration and conservation [1, 2]. The U.S. Bureau of Land Management (BLM), for example, recommends using a landscape approach to identify the most appropriate combination of mitigation measures across all relevant geographic scales, in order to provide the most benefit to resources on public lands impacted by human activities or other change agents such as wildfire or climate change [3]. The National Park Service is also using large landscape conservation approaches and collaboration with multiple public and private groups to enhance the values of national trails, rivers, and other areas [4]. In the past, land managers have been challenged in effectively applying landscape approaches by the unavailability of data at the appropriate spatial and temporal scales. However, advancements in computing and geospatial analysis over the past decade have allowed agencies to synthesize more geospatial data on resource condition and trends at sufficient resolutions and spatial extents to better understand management priorities over broad scales. For example, the BLM began conducting Rapid Ecoregional Assessments (REAs) to map and quantify the status and trends of natural resources within an ecoregion [5, 6]. Information from these REAs have been used by the BLM and other Federal agencies to evaluate the potential impacts of proposed human development in a regional context and identify conservation opportunities to offset these impacts [7–9].

The landscape approach to conservation planning often involves the development and synthesis of broad-scale indicators of resource distribution, status, and condition [10]. One trend among conservation planners is to use a landscape measure of ecological integrity to better understand human-caused impacts in the environment and identify areas of potential conservation value [11–13]. In these applications, ecological integrity is defined as the ability of an ecological system to support and maintain a community of organisms and habitats with structure and composition, diversity, and functional organization similar to the system’s natural habitat [14, 15]. Areas of high integrity are capable of naturally recovering from disturbance (depending on the nature of disturbance) and are relatively unimpaired by human activities. Human land uses affect ecological integrity, through factors such as vegetation and habitat loss and alteration, hydrologic alterations, and introduction and spread of invasive species. Models of landscape ecological integrity, therefore, have been largely focused on quantifying the human presence in the environment [11, 16, 17] and generally rely on indicators derived from human development location data to quantify and map ecological integrity. The ecological consequences of human land uses differ based on the permanence of the activity and the degree of modification from natural conditions [18, 19]. Previous landscape modeling efforts have focused on the influence of human land uses on ecosystems by mapping landscape human development indicators (or human footprint [20, 21]).

While approaches to evaluate and map the intensity of human activity provide general indicators of ecological integrity across landscapes to inform land management decisions, challenges with these approaches arise when considering the implications of other landscape-scale processes that may not be directly linked to human development [10, 22]. Although most landscape integrity models rely on measures of the degree of human modification in the environment, the presence and magnitude of the human footprint is only one indicator of ecological integrity. Other landscape indicators of ecological integrity may include compositional attributes, such as species richness, and other disturbance processes, such as climate change and the return interval of wildfire [12, 15, 22, 23]. Previous modeling efforts have been limited by the lack of available data for other indicators of integrity but advancements in data collection, synthesis, and assessment have created opportunities to more comprehensively characterize ecological integrity using approaches that integrate indicators of other ecological integrity attributes (e.g., species richness and vegetation departure).

In this paper we present a framework to quantify and map a Landscape Integrity Index (*LII*) as a landscape indicator of ecological integrity. This modeling framework builds upon previously-published methods to map the degree of human modification in the environment (i.e., the human footprint). Our framework is based on landscape measures of human influence and modification, biodiversity (e.g., species richness), and other measures of landscape change (e.g., vegetation departure). We focused on developing a computationally simple yet robust approach to model *LII* that incorporates several components of ecological integrity. We discuss the process by which this *LII* model was developed and present how land managers may apply this *LII* modeling framework to inform regional conservation planning on public lands in a region of southern Colorado and northern New Mexico, USA. Although we present model results for a specific region in the western USA, the framework can be replicated elsewhere where sufficient data exist.

## Materials and methods

### Study area

**Fig 1:**
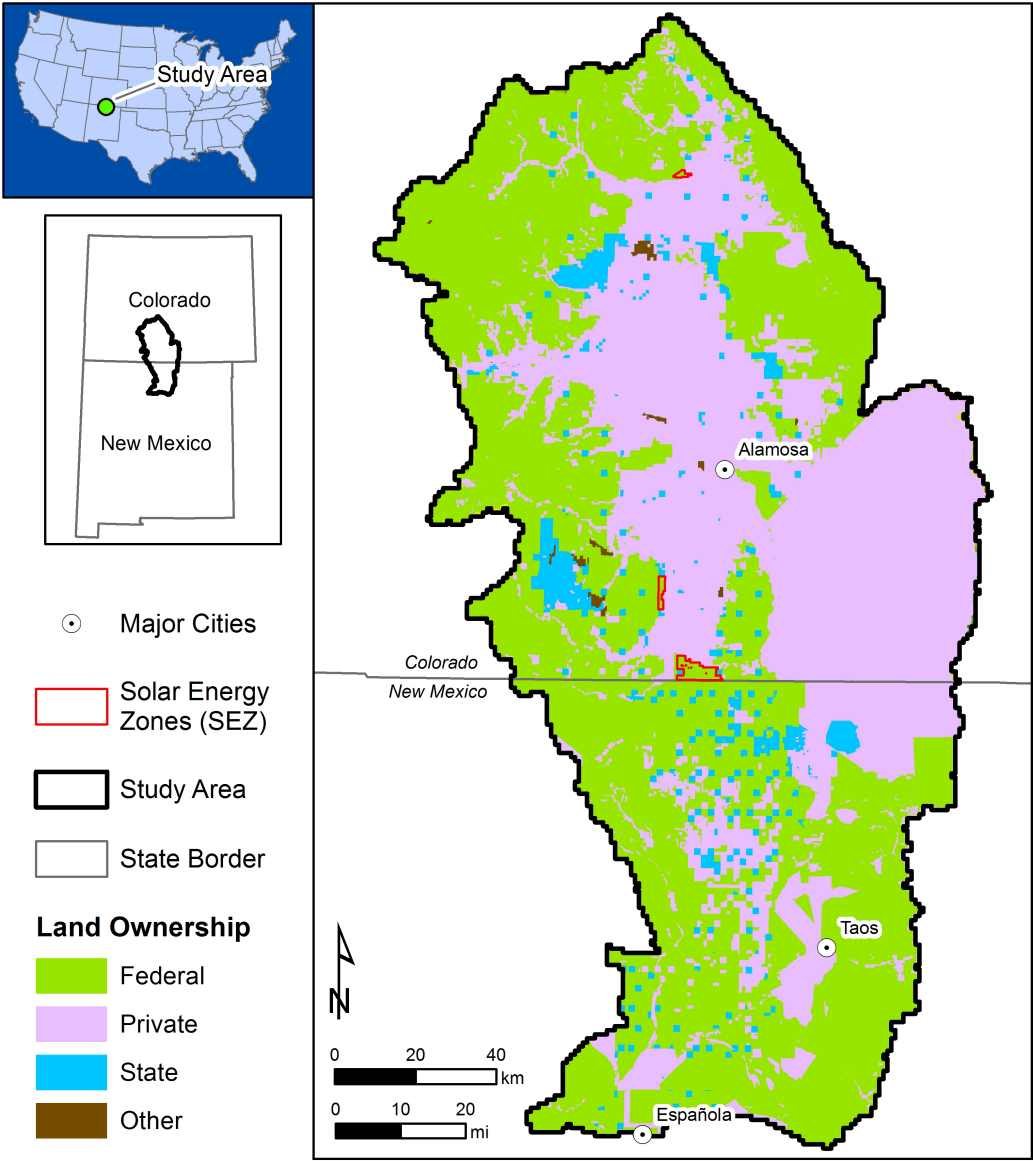
The San Luis Valley–Taos Plateau study area of southern Colorado and northern New Mexico.

The study area in which we applied this landscape modeling framework was the San Luis Valley of southern Colorado and the Taos Plateau of northern New Mexico (hereafter “study area”, Fig 1), as defined by the San Luis Valley and Taos Plateau Level IV Ecoregions [24]. Approximately 53% of the land area in this region is publicly managed by the U.S. Federal government (Fig 1). The 25,300 km^2^ study area is bounded by two dominant mountain ranges in the region: the Sangre de Cristo Mountains in the east, and the San Juan Mountains in the west. Elevations within the study area range from approximately 1,500 m to 4,270 m. Human occupation of the study area is primarily rural in nature, with over 20% of the area utilized for agriculture or livestock grazing. The largest population centers are Alamosa, CO (population: 9,500) and Española, NM (population: 10,500).

This region was chosen on the basis of recent landscape planning efforts implemented by the U.S. Department of the Interior’s Bureau of Land Management (BLM) to evaluate regional compensatory mitigation opportunities for utility-scale solar energy development on public lands [9]. Approximately 15% of the study area is managed by the BLM. The BLM has identified three Solar Energy Zones (SEZs) in the study area as priority areas for future utility-scale solar energy development (Fig 1). The SEZs are located on 54 km^2^ (13,300 acres) of arid shrubland vegetation at elevations between 2,000 m and 2,400 m (6,560 ft and 7,875 ft).

### Modeling the degree of human influence

**Fig 2:**
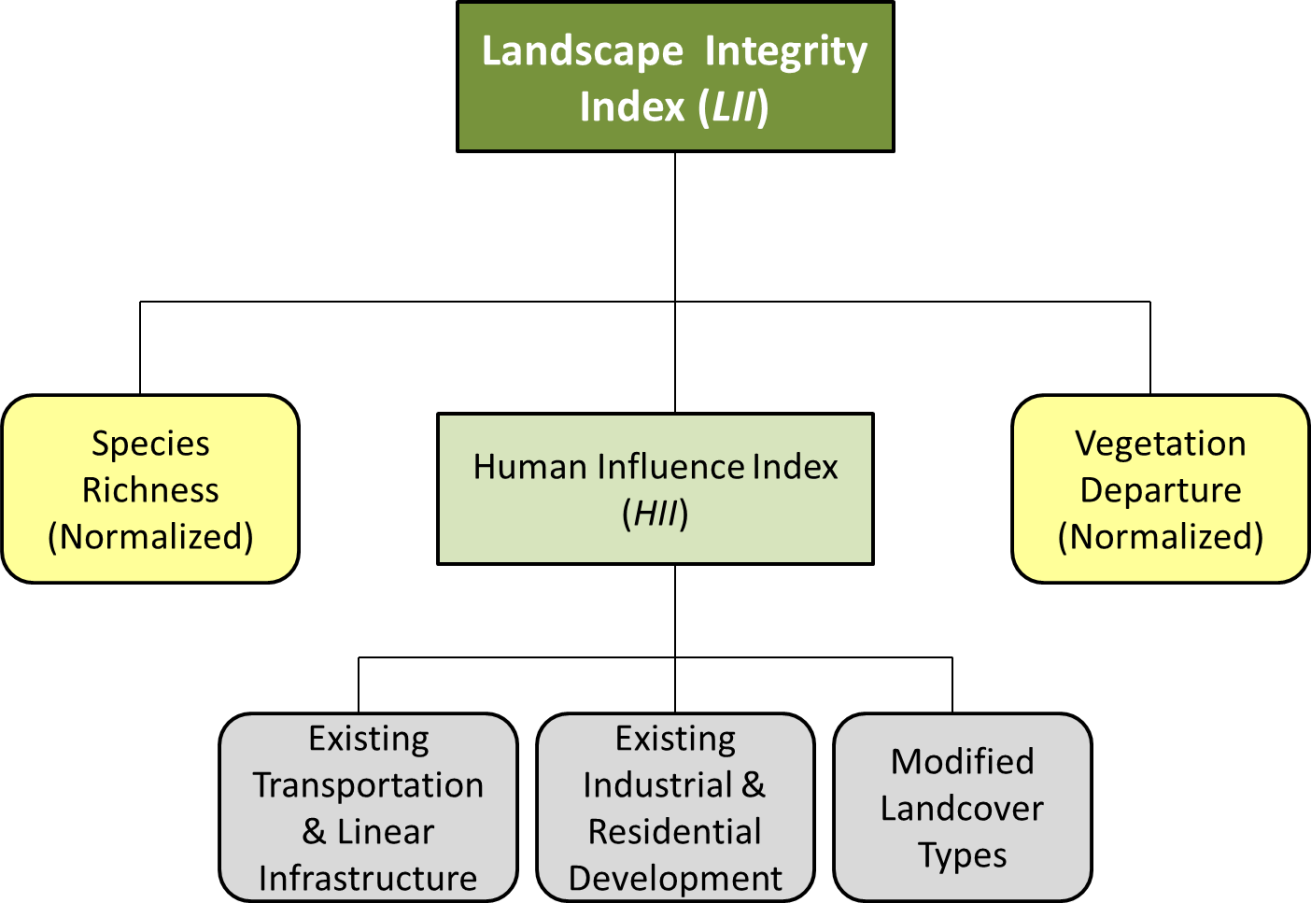
Framework for modeling the Landscape Integrity Index (*LII*). Refer to Table 1 for spatial inputs and parameters for the Human Influence Index (*HII*). Refer to Methods section and Table 1 for data sources and modeling approach.

Our framework for quantifying and mapping *LII* builds on previous approaches that focus on mapping and quantifying the degree of human modification across landscapes [11, 16, 17]. At the center of our framework, we developed a Human Influence Index (*HII*), based on methods used by Woolmer et al. [16] and Sanderson et al. [20] and similar to the approach described by Theobald [11], to characterize the extent and intensity of human development across the study area. We then incorporated measures of vegetation departure and species richness across the landscape to develop the *LII* (Fig 2). We included 16 spatial datasets as inputs to the *HII*, representing various levels of human land use in the study area (Table 1). Our approach involved the use of a composite scoring system to parameterize the following for each input: (1) site impact score–the assumed intensity of the human land use (value range from 0.0 to 1.0) and (2) the presumed distance of influence–the maximum distance at which the input dataset was assumed to influence ecological integrity. After processing the data to meet the input definitions identified in Table 1, we calculated the Euclidean distance of each input layer. The Euclidean distance geoprocess produced a continuous rasterized output (90m resolution) for each input dataset across the study area where each cell value represented the distance (meters) to the nearest mapped feature.

**Table 1:** Spatial data inputs and parameterization of the Human Influence Index (*HII*) for the San Luis Valley–Taos Plateau study area^1^.

Human Land Use or Impact Factor	Site Impact Score ^2^	Distance of Influence (m) ^3^	Distance-Decay Function ^4^	Data Sources^5^
**Transportation**	** **	** **	** **	
Primitive roads (e.g., dirt roads and trails)	0.75	500	linear	1
Local roads	0.3	1500	logistic	1
Major highways	0.015	4000	logistic	1
**Urban and Industrial Development**	** **		** **	
Low density development (including rural development)	0.6	1000	logistic	2
Medium density development	0.35	2000	logistic	2
High density development	0.015	4000	logistic	2
Communication towers	0.6	200	linear	3
Powerlines and utility lines	0.6	200	linear	4, 5
Mines and oil & gas well pad locations	0.2	1000	logistic	6, 7
Urban Polygons (U.S. Census Bureau)	0.015	4000	logistic	8
High Impervious Surfaces (National Land Cover Database)	0.3	1000	logistic	9
**Managed and Modified Land Cover**	** **	** **	** **	
Low agriculture and invasives (ruderal forest, recently burned, recently logged, etc.)	0.7	500	linear	2
Pasture (landcover)	0.7	500	linear	2
Grazing allotment polygons	0.7	500	linear	10
Introduced vegetation	0.6	500	linear	2
Cultivated agriculture	0.35	2000	linear	2

1 Modeling approach and parameters are adopted from previous landscape modeling efforts [11, 17].

2 Site Impact Score ranges between 0 and 1 and provides an indication of presumed ecological stress or impact. Lower values (closer to 0) indicate a greater site impact. Values adopted from previous modeling efforts [11, 16, 17].

3 Distance of influence is the minimum distance at which intactness values approach 1.0. Values adopted from previous modeling efforts [17].

4 Distance decay functions for impacting factors with low or moderate relative levels of stress were evaluated with linear or logistic functions. Distance decay functions for impacting factors with high relative levels of stress were evaluated with logistic functions.

5 Data Sources: 1—TIGER Roads (https://www.census.gov/geo/maps-data/data/tiger.html); 2—LANDFIRE Existing Vegetation Types (https://www.landfire.gov/evt.php); 3—Federal Communications Commission cellular towers (http://www.arcgis.com/home/item.html?id=e1df814d7e864791ad0e920f1d37c13d); 4—Department of Homeland Security electric power transmission lines (https://hifld-dhs-gii.opendata.arcgis.com/datasets/37654d07acfc45689b82fbfc64031d40_0); 5—Bureau of Land Management utility lines for the San Luis Valley–Taos Plateau Ecoregion (https://landscape.blm.gov/SLV_2013_layerpackages/SLV_Utility_Lines.lpk); 6—Bureau of Land Management mines for the San Luis Valley–Taos Plateau Ecoregion (https://landscape.blm.gov/SLV_2013_layerpackages/SLV_Mines_Point.lpk); 7—Bureau of Land Management oil and gas lease areas for San Luis Valley–Taos Plateau Ecoregion (https://landscape.blm.gov/SLV_2013_layerpackages/SLV_BLM_Oil_Gas_Lease_Poly.lpk); 8—U.S. Census Bureau urban areas (https://www.census.gov/geo/maps-data/data/cbf/cbf_ua.html); 9—National Land Cover Database Impervious Surfaces (https://www.mrlc.gov/nlcd2011.php); 10 –Bureau of Land Management grazing allotments for the San Luis Valley–Taos Plateau Ecoregion (https://landscape.blm.gov/SLV_2013_layerpackages/SLV_Allotments_BLM_Poly.lpk).

Similar to previous modeling approaches (e.g., [11, 17]), we used a relative scoring system to parameterize site impact scores within a normalized range of 0.0 (high human modification) to 1.0 (low human modification). Site impact scores in our *HII* model were adopted from relative site impact scores developed for previously published models [11, 16, 17] (Table 1). For example, recently logged areas were given a relatively high site impact score (0.7) compared to cultivated agriculture (0.35) and high-intensity urban development (0.015).

**Fig 3:**
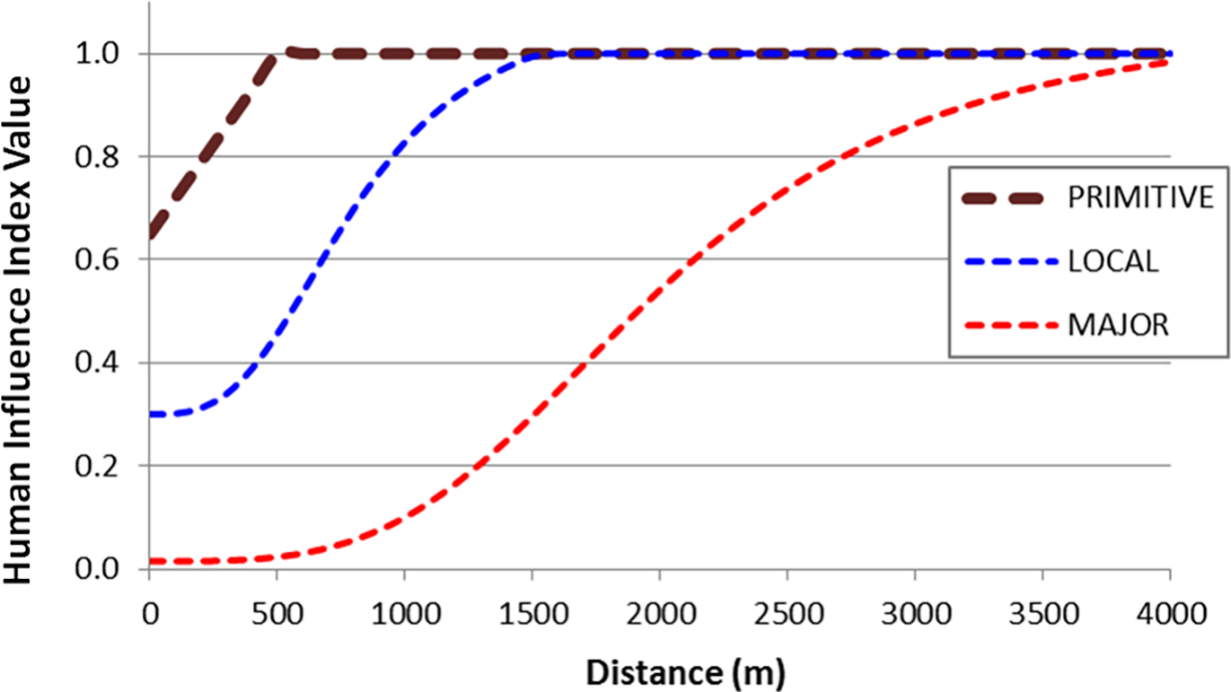
Distance decay functions for the three types of roadways (primitive, local, and major) evaluated in the development of the Human Influence Index (*HII*). Refer to Table 1 for model parameterization.

Proximity to human modification is a fundamental driver of landscape models of ecological integrity [11]. Habitat quality and use by wildlife generally decreases with proximity to human activities. For example, Rowland et al. [25] found there was a measurable decline in elk habitat use up to 1.8 km (1.1 mi) away from roadways. Most effects to wildlife have been reported to be within 4 km (2.5 mi) of human developments ([25–28]; but see [29]). We therefore used this distance (4 km) as the maximum distance of influence in the *HII* model. We developed distance decay functions for all input layers that expressed the relationship between ecological impact and distance from the input feature. These distance decay curves varied based on the presumed degree of impact for each input dataset. Distance decay functions for input datasets that have higher site impacts (e.g., high-intensity urban developments) were parameterized with logistic functions whereas distance decay functions for input datasets that have lower site impacts (e.g., areas of grazing) were parameterized with linear functions (Table 1). An example of how *HII* was modeled for three types of roadways, with various levels of presumed ecological impact, is provided in Fig 3.

We used ArcGIS v. 10.3 raster calculator to model *HII* for each of the 16 input datasets (Table 1) by applying the individual distance decay function to the Euclidean distance raster for each input layer. This resulted in 16 individual *HII* raster models within standardized 90-m pixels across the study area (the finest resolution of all inputs). Because all *HII* values were represented along the same continuous scale (0 to 1), we overlaid all 16 *HII* models and used cell statistics to compute the minimum *HII* value as the composite measure of *HII* in the final model. The final *HII* model was calculated within the native 90-m raster; this *HII* output model was later used to model *LII* (Fig 2).

### Species richness and vegetation departure

Our approach to model *LII* incorporated the *HII* along with two additional indicators of ecological integrity (Fig 2): a composite measure of species richness for terrestrial vertebrates and vegetation departure (the measured change in current mapped vegetation communities from modeled historic vegetation communities). Wurtzebach and Schultz [22] emphasized the importance of incorporating additional datasets such as these as indicators of compositional and functional attributes of ecological integrity. Species richness has been regarded as an indicator of ecological composition (i.e., areas of greater species richness have higher ecological integrity). We obtained habitat suitability models from the Southwest Regional Gap Analysis Program (SWReGAP) [30] for all terrestrial wildlife vertebrates that occurred within the study area and for which SWReGAP models were available. This totaled 137 species-specific habitat suitability models (S1 Table). Each species distribution model consisted of a 240-m integer raster dataset depicting categorical levels of habitat suitability. To generate a model of species richness across the study area, we converted all 137 of these species-specific models to binary datasets, where values of zero (0) indicated areas of no habitat suitability and values of one (1) indicated areas of suitable habitat. To calculate species richness, all 137 binary datasets were then summed using cell statistics in ArcGIS v 10.3 to provide a single aggregated measure of the number of species with suitable habitat within each 240-m pixel.

We normalized terrestrial vertebrate species richness values within the 240-m pixels along the same low-high gradient (ranging between 0 and 1) as the *HII*, based on the minimum and maximum species richness values across all pixels. In this way, the scale of species richness model was identical to the *HII* scale (i.e., values closer to 0 indicated low species richness; values closer to 1 indicated high species richness). The normalized 240-m raster of species richness was later used to model *LII* (Fig 2).

We obtained geospatial data on vegetation departure from the LANDFIRE Program [31]. The LANDFIRE Program is a shared program between the wildland fire management programs of the U.S. Department of Agriculture Forest Service (USFS) and U.S. Department of the Interior (DOI), providing landscape scale geospatial products to support cross-boundary planning, management, and operations. The Vegetation Departure (VDEP) data layer produced by the LANDFIRE Program quantifies the departure between current vegetation conditions and reference vegetation conditions within 30-m raster pixels according to the methods outlined in the Interagency Fire Regime Condition Class Guidebook [32]. These vegetation departure values represent changes to species composition, structural stage, and canopy closure that may be due to factors such as changes in human land use; wildfire frequency, magnitude, and extent; and climate change. As such, we have incorporated this dataset in the *LII* model as an added measure of ecological function and composition. Downloaded VDEP values ranged from 0 to 100 to depict the amount current vegetation has departed from simulated historical vegetation reference conditions. We used the normalized inverse VDEP values along the same low-high gradient as the *HII* (ranging between 0 and 1). In this way, the scale of normalized inverse VDEP values was identical to the *HII* scale (i.e., values closer to 0 indicated high vegetation change from historic conditions; values closer to 1 indicated little vegetation change from historic conditions). The normalized 30-m raster of vegetation departure was later used to model *LII* (Fig 2).

### The Landscape Integrity Index

All normalized raster models of *HII*, terrestrial vertebrate species richness, and VDEP were resampled to uniform 90-m raster grids with equal extents. We computed *LII* by calculating the average of all overlapping 90-m pixel values within 1 km moving windows (Fig 2). Equal weight was given to the three modeled parameters (species richness, *HII*, and VDEP). We used ArcGIS v 10.3 with the Spatial Analyst Extension to conduct the moving window analysis. The resulting map depicted landscape integrity across the study area with *LII* scores ranging from 0 to 1 for each 90-m pixel, based on 1 km moving widow averages surrounding each pixel. *LII* values closer to 0 represent low landscape integrity; values closer to 1 represent higher landscape integrity. We summarized landscape integrity across the study area and evaluated how *LII* scores differed by land ownership type through a one way Analysis of Variance (ANOVA).

We evaluated our landscape integrity model in two ways. First, at 100 randomly selected points across the study area, we compared *LII* scores to modeled human footprint scores computed by Leu et al. [21]. Because the human footprint model produced by Leu et al. [21] focused solely on anthropogenic disturbances and our *LII* model incorporated additional spatial indicators of ecological integrity that were not directly related to human land use (e.g., species richness, invasive species, vegetation departure), we expected there to be differences between the models that would result in a weak or moderate correlation.

Second, we compared *LII* values between currently protected and unprotected areas according to the Protected Areas Database [33]. We queried the Protected Areas Database to select only those areas with status codes 1 and 2 to represent the protected areas in this study, as these areas are highly protected lands managed to maintain natural qualities [34]. We then determined *LII* values at 50 randomly selected locations within protected and unprotected areas (100 total locations) and compared *LII* between these protection levels using a Welch’s two sample t-test. All statistical analyses were performed in R 3.2.3 [35].

### Applying the landscape integrity model in conservation planning

We highlight example applications of the *LII* to inform land management options as mitigation for anticipated solar energy development on the SEZs in the study area. The BLM implements a landscape approach in the mitigation hierarchy to avoid, minimize, and compensate for the impacts of land use decisions, including solar development [9]. Among other applications, this landscape approach to mitigation planning involves the use of broad-scale data and indicators to identify and evaluate conservation priorities that may be recommended as preferred compensatory mitigation actions. Landscape-scale mitigation planning involves considerations for several resources and other criteria, including offsets for ecological, cultural, hydrological resources and human dimensions (e.g., socioeconomics). Here, we illustrate how the *LII* model may be used in BLM’s landscape approach to develop empirically-based mitigation priorities based on two ecological conservation goals: (A) maintaining ecological connectivity between protected priority areas of conservation and (B) increasing the size of the protected area network based on landscape integrity.

First, we used the *LII* model to develop coarse-filter naturalness-based connectivity models that link the ecological priority areas that are currently protected to preserve biodiversity. There is a growing interest among conservation planners in identifying conservation corridors based on broad-scale measures of ecological integrity or “naturalness” (e.g., [34, 36]). To identify conservation opportunities that directly offset the impacts of solar development on the SEZs, we limited our evaluation to arid shrubland systems that are similar to those located on the Solar Energy Zones. To do this, we used LANDFIRE Biophysical Settings [37] to identify and extract the extent of the arid shrubland system in which the three BLM SEZs have been designated. We then used the U.S. Protected Areas Database (PAD-US) [33] to identify all ecological priority areas within this extent that are protected for biodiversity (GAP status 1 and 2) [11, 34]. Using the *LII* model as the resistance layer, we then applied the Linkage Mapper toolbox for ArcGIS [38] to map least-cost corridors that linked ecological priority areas. Similar to Krosby et al. [36], we identified corridor networks based on the highest-value 30% of the land area outside the ecological priority areas. We summarize the results of these corridor models as potential mitigation for solar development on the BLM SEZs.

Second, we used the *LII* model to identify high-integrity lands adjacent to current priority areas that could be afforded protection and expand the protected areas network. In this approach, we focused on lands adjacent to current ecological priority areas so as to maintain ecological connectivity. We used the upper 30% of *LII* values (where *LII* >0.70) as the threshold in identifying high-integrity lands under this approach. We summarize the results of this analysis to identify conservation opportunities as potential mitigation for solar development on the BLM SEZs.

## Results

### The landscape integrity model

**Fig 4:**
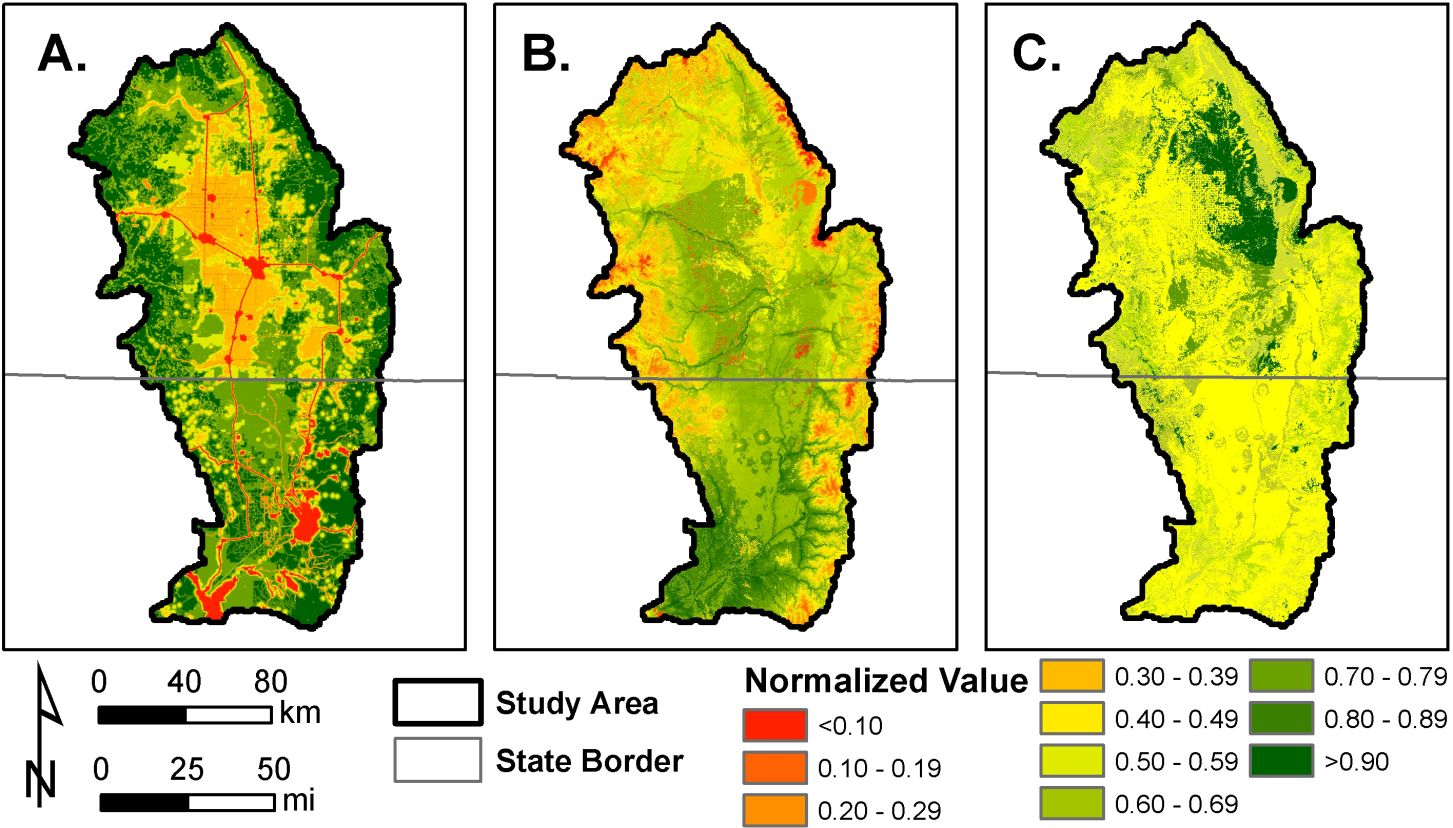
Normalized model values for the (A.) Human Influence Index (*HII*), (B.) species richness, and (C.) vegetation departure for the San Luis Valley–Taos Plateau study area. These normalized values, ranging between 0 and 1, were incorporated into the final Landscape Integrity Index (*LII*) (Fig 5).

**Fig 5:**
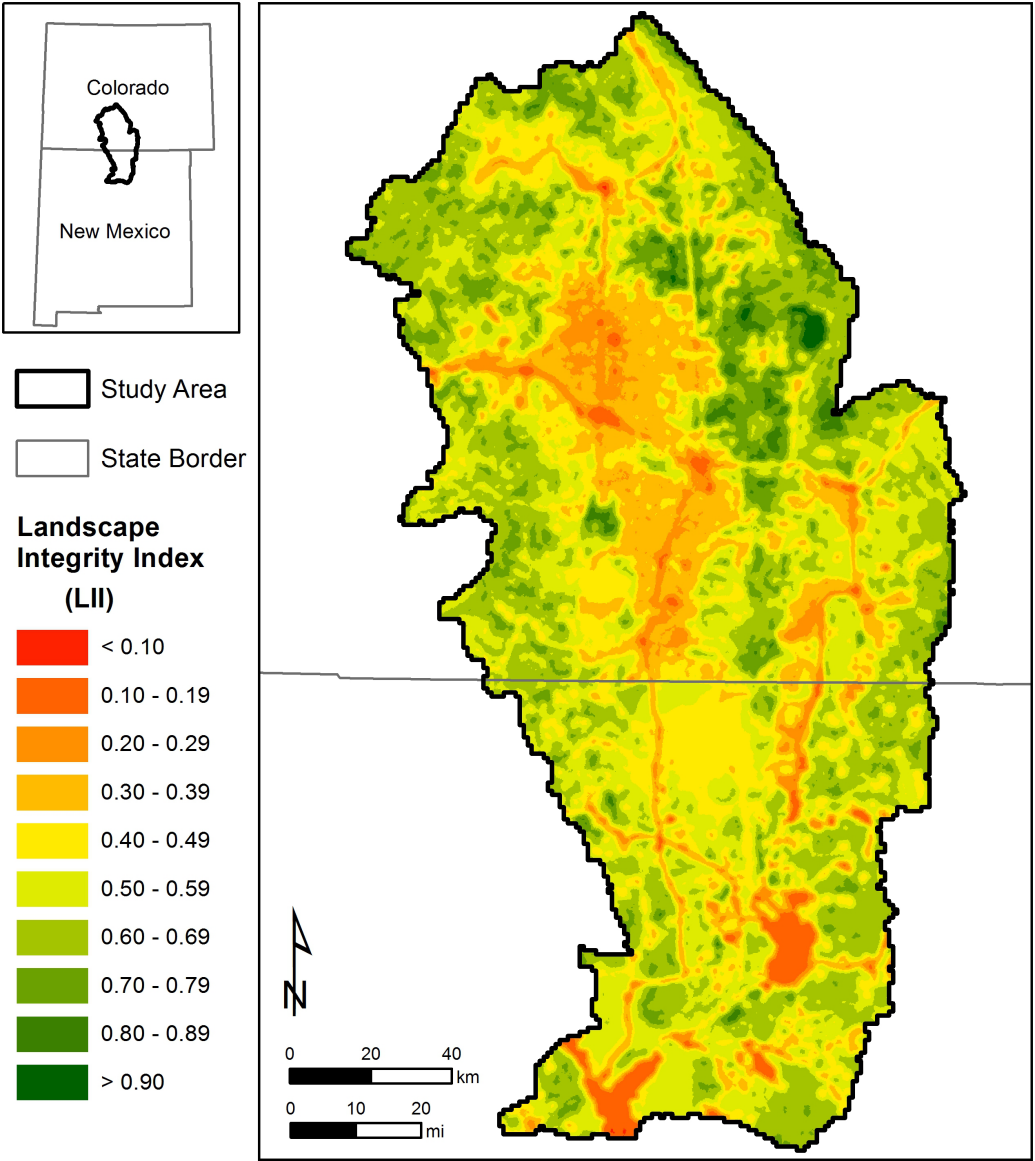
Final Landscape Integrity Index (*LII*) model for the San Luis Valley–Taos Plateau study area, calculated as the 1-km moving window mean of intermediate models of the Human Influence Index (*HII*), species richness, and vegetation departure (Fig 4).

**Fig 6:**
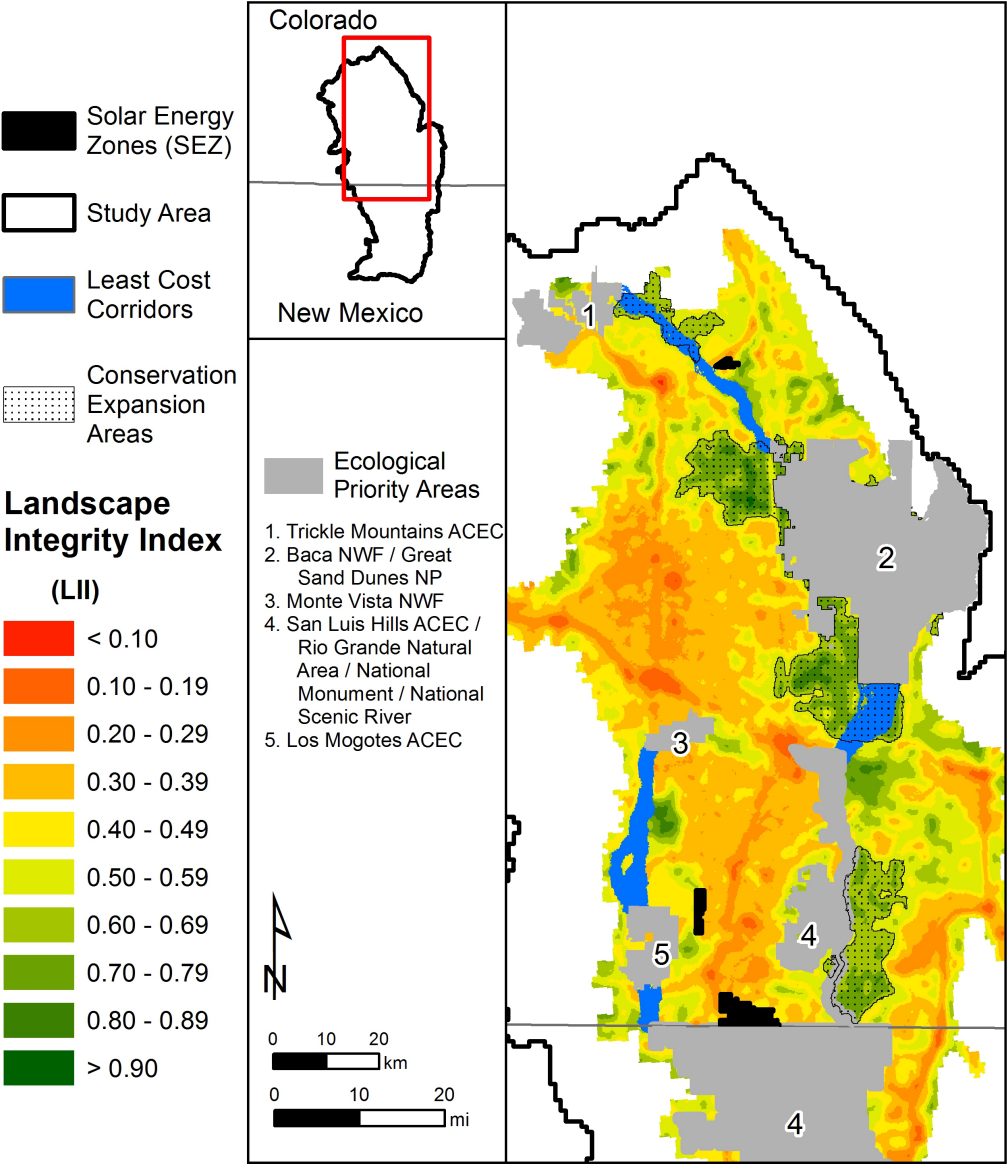
Landscape integrity within the extent of the grassland and shrubland system where solar energy development is anticipated within the Bureau of Land Management’s Solar Energy Zones. Five ecological priority areas within this grassland and shrubland system are also shown.

Overlapping normalized values of *HII*, species richness, and vegetation departure were averaged to produce the *LII* (Figs 4 and 5). Patterns of human settlement and human-induced landscape change were observable in the final *LII* model (Fig 5), such as populated areas and major roadways (Fig 4). The final *LII* results approximated a normal distribution with a mean of 0.530, indicating a moderate level of landscape integrity throughout the region. Approximately 12% of the region had *LII* values >0.75, whereas approximately 10% of the region is characterized by *LII* values <0.25. The remaining 78% of the region occurs in areas of intermediate *LII* values between 0.25 and 0.75. Areas of greatest landscape integrity were located in the vicinity of the Baca National Wildlife Refuge and Great Sand Dunes National Park in the eastern portion of the study area (Fig 6). These areas have been managed to minimize human intrusion and restore ecological function. Areas of lowest landscape integrity occurred in areas where the human footprint was greatest, particularly near urban areas, major roadways, and areas of row crop agriculture.

There was a significant difference in *LII* values by land ownership (Table 2; F_2,147_ = 14.78; P<0.001). Lands managed by Federal government agencies contained average *LII* values of 0.574 (±0.114 SD). Private lands, on the other hand, contained average *LII* values of 0.472 (±0.155 SD). These *LII* values on private lands were significantly lower than *LII* values on Federal- or State-managed lands because most of privately-owned lands were utilized by humans (e.g., residences, urbanization, agriculture) (Table 2).

**Table 2:** Summary of Landscape Integrity Index (*LII*) scores by land ownership type.

Ownership	Size (km^2^) and Percent of Study Area (in Parentheses)	Average *LII* Score (Standard Deviation)	Analysis of Variance ^1^
Federal	13,500 (53%)	0.574 (0.114)	a
Private	10,600 (42%)	0.472 (0.155)	b
State	1,100 (4%)	0.544 (0.115)	a
Other	100 (1%)	0.517 (0.089)	―

1 There was an overall significant difference in Landscape Integrity Index values among the land ownership types (one way Analysis of Variance; F2,147 = 14.78; P<0.001). Differences among ownership types are denoted alphabetically (a-b), based on Tukey HSD post hoc comparisons (α = 0.01). Other ownership types were excluded from statistical analysis due to the small size of these areas.

We found a weak but significant correlation between randomly selected *LII* scores and the human footprint model developed by Leu et al. [21] (r = -0.492; P<0.001). We also found that *LII* scores within protected areas were greater than *LII* scores in unprotected areas (t_98_ = 2.750; p = 0.007). On average, the *LII* value within protected areas was 0.611 (+/- 0.022 SE), whereas the *LII* value within unprotected areas was 0.524 (+/- 0.023 SE). Rural activities such as agriculture and livestock grazing were the primary human land use drivers of ecological integrity across the study area. Because these activities may have historically occurred in both protected and unprotected areas (i.e., prior to sites becoming designated as protected areas), the ecological effects of these activities may still be measurable on the landscape through datasets such as vegetation departure (Fig 4).

### Application of the landscape integrity model in conservation planning

We identified 5 ecological priority areas within the basin grassland and shrubland systems in the study area based on the protected areas database where GAP Status was equal to 1 or 2 [33] (Fig 6). Using the *LII* model as a coarse-filter resistance layer, we developed 4 habitat connectivity corridors to link these priority areas as a function of the degree of “naturalness” between these areas. These least-cost corridors represent the paths of highest landscape integrity that link priority areas. The entire footprint of these corridors totals over 325 km^2^, which is more than 6 times greater than the BLM SEZs that would be disturbed by solar energy development (Fig 6).

We also found that the *LII* model could be used to expand the protected areas network by identifying high integrity lands adjacent to current ecological priority areas that could be afforded protection. Using the upper 30% of *LII* values, we were able to identify over 900 km^2^ of high integrity adjacent land that could be considered to expand the protected areas network (Fig 6). The size of these expansion areas is nearly 17 times greater than the BLM SEZs.

## Discussion

Human-induced landscape changes have been identified as one of the greatest threats to biodiversity [39, 40]. Areas where human populations have increased are facing increasing difficulty in minimizing habitat loss, preserving biodiversity, and maintaining ecological functions and ecosystem services (i.e., “the environmentalist’s paradox”) [23, 41, 42]. This is particularly true for portions of the western United States that have experienced relatively rapid rates of human modification in the past century. The human footprint is prevalent throughout our study area in Colorado and New Mexico, with approximately 20% of the study area characterized by some form of human development (e.g., urban development, roads, agriculture, etc). Today, over 50% of the study area is located within 4 km of human development.

Efforts to quantify and map human developments have provided reasonable coarse-filter indicators of ecological integrity by mapping the degree of “naturalness” across landscapes [e.g., 11, 17]. However, human development datasets alone may not be sufficient landscape indicators of all attributes of ecological integrity. Other landscape indicators of ecological integrity that we have included in our *LII* model include compositional attributes, such as species richness (biodiversity) and vegetation departure, which could be used as a measure of ecological departure from the historic conditions based on human activity or other environmental drivers such as climate change, invasive species, or wildfire [22, 43]. As expected, our modeled *LII* values were greater within areas already protected for biodiversity, where human disturbance has been minimized and habitats have been managed to maintain natural processes. On average, *LII* values within protected areas were about 16.6% greater than *LII* values outside protected areas. We also noted a moderate correlation between our LII values and modeled estimates of the human footprint by Leu et al. [21]. This finding could be due to the fact that our *HII* model is another representation of the human footprint that is highly correlated with the footprint model by Leu et al. [21] (r = -0.735; p < 0.001). We therefore expected our resulting *LII* model to retain some correlation with the human footprint.

Designating and maintaining an interconnected network of protected areas that are of high ecological value is one of the most important means to conserve biodiversity in the face of ongoing outside environmental changes, such as those due to climate change, human development, and invasive species [36, 44]. We examined two coarse-filter applications of the *LII* model to inform regional conservation planning that focused on approaches to increase the size and connectivity of the protected area network as mitigation for anticipated future utility-scale solar energy development in the region.

Between these two approaches, we identified over 1,000 km^2^ of high integrity areas in the region outside of the existing protected areas network that could be valuable in either linking or expanding the size of existing ecological priority areas. The connectivity and expansion areas we identified are over 17 times larger than the BLM SEZs, which should provide many opportunities to meet various biodiversity conservation objectives to mitigate anticipated ecological impacts of solar energy development within the SEZs and other future human land use changes in the region. Similar studies have used general indicators of landscape integrity to identify opportunities to improve biodiversity protection by increasing the size and connectedness of the protected areas network [11]. Coarse-filter approaches to landscape planning such as these that use general indicators of landscape integrity have been found to be highly efficient compared to fine-filter (e.g., species-specific) approaches [36, 45]. Like other coarse-filter approaches, our example approaches can be modified to identify conservation opportunities based on different ecological or administrative objectives. The *LII* modeling framework is also temporally dynamic such that new or updated spatial datasets may be incorporated as they are developed in the future (e.g., every 3–5 years). This functionality would help land managers and conservation planners better understand status and trends in landscape integrity.

Our study highlights an ecologically-driven framework to conservation planning. However, landscape approaches to conservation planning often require the need to balance tradeoffs among different and often conflicting land uses (e.g., cultural, ecological, and visual concerns) [46, 47]. Our *LII* model framework may assist other landscape-scale structured multi-criterion support frameworks by providing input on ecological values and status/trends.

## Supporting information

S1 Table List of 137 species with SWReGAP habitat models included in the species richness model. (DOCX)
