# Supplementary material for: Development of a landscape integrity model framework to support regional conservation planning
Source: PLoS One. 2018 Apr 3;13(4):e0195115. doi: 10.1371/journal.pone.0195115 (PMC5882122; doi:10.1371/journal.pone.0195115)
Supplement: S1 Table — (DOCX) [file pone.0195115.s001.docx]

**Supplemental Table 1. List of 137 Species with SWReGAP Habitat Models Included in the Species Richness Model.**

| **N** | **IT IS Code** | **Taxonomic Group** | **Common Name** |  | **N** | **IT IS Code** | **Taxonomic Group** | **Common Name** |
| --- | --- | --- | --- | --- | --- | --- | --- | --- |
| 1 | 173429 | Amphibian | Couch's Spadefoot Toad |  | 39 | 178625 | Bird | Gray Catbird |
| 2 | 173484 | Amphibian | Great Plains Toad |  | 40 | 177884 | Bird | Great Horned Owl |
| 3 | 173663 | Amphibian | Jemez Mountains Salamander |  | 41 | 177836 | Bird | Greater Roadrunner |
| 4 | 173443 | Amphibian | Northern Leopard Frog |  | 42 | 179310 | Bird | Green-Tailed Towhee |
| 5 | 173448 | Amphibian | Plains Leopard Frog |  | 43 | -2 | Bird | Gunnison Sage-Grouse |
| 6 | 173491 | Amphibian | Red-Spotted Toad |  | 44 | 179884 | Bird | Hepatic Tanager |
| 7 | 173592 | Amphibian | Tiger Salamander |  | 45 | 554256 | Bird | Horned Lark |
| 8 | 173482 | Amphibian | Western Toad |  | 46 | 179191 | Bird | House Finch |
| 9 | 209400 | Reptile | Bullsnake (Gopher Snake) |  | 47 | 176520 | Bird | Killdeer |
| 10 | 174017 | Reptile | Chihuahuan Spotted Whiptail |  | 48 | 178260 | Bird | Ladder-Backed Woodpecker |
| 11 | 174238 | Reptile | Coachwhip |  | 49 | 176656 | Bird | Least Sandpiper |
| 12 | 209247 | Reptile | Common Kingsnake |  | 50 | 178196 | Bird | Lewis's Woodpecker |
| 13 | 174202 | Reptile | Glossy Snake |  | 51 | 178515 | Bird | Loggerhead Shrike |
| 14 | 174187 | Reptile | Milk Snake |  | 52 | 177932 | Bird | Long-Eared Owl |
| 15 | 173956 | Reptile | Side-Blotched Lizard |  | 53 | 175613 | Bird | Merlin |
| 16 | 174319 | Reptile | Western Rattlesnake |  | 54 | 554385 | Bird | Mountain Chickadee |
| 17 | 175622 | Bird | American Kestrel |  | 55 | 176522 | Bird | Mountain Plover |
| 18 | 178979 | Bird | American Redstart |  | 56 | 177125 | Bird | Mourning Dove |
| 19 | 174684 | Bird | American White Pelican |  | 57 | 178154 | Bird | Northern Flicker |
| 20 | 178316 | Bird | Ash-Throated Flycatcher |  | 58 | 175300 | Bird | Northern Goshawk |
| 21 | 175420 | Bird | Bald Eagle |  | 59 | 177942 | Bird | Northern Saw-Whet Owl |
| 22 | 175144 | Bird | Barrow's Goldeneye |  | 60 | 175590 | Bird | Osprey Pandion |
| 23 | 178119 | Bird | Belted Kingfisher |  | 61 | 175604 | Bird | Peregrine Falcon |
| 24 | 178636 | Bird | Bendire's Thrasher |  | 62 | 174505 | Bird | Pied-Billed Grebe |
| 25 | 177997 | Bird | Black Swift |  | 63 | 179205 | Bird | Pine Grosbeak |
| 26 | 554382 | Bird | Black-Capped Chickadee |  | 64 | 175603 | Bird | Prairie Falcon |
| 27 | 174832 | Bird | Black-Crowned Night-Heron |  | 65 | 175350 | Bird | Red-Tailed Hawk |
| 28 | 179395 | Bird | Black-Throated Sparrow |  | 66 | 175905 | Bird | Ring-Necked Pheasant |
| 29 | 179440 | Bird | Brewer's Sparrow |  | 67 | 175373 | Bird | Rough-Legged Hawk |
| 30 | 177946 | Bird | Burrowing Owl |  | 68 | 179870 | Bird | Ruby-Crowned Kinglet |
| 31 | 174803 | Bird | Cattle Egret |  | 69 | 179402 | Bird | Sage Sparrow |
| 32 | 554027 | Bird | Clark's Grebe |  | 70 | 176177 | Bird | Sandhill Crane |
| 33 | 555544 | Bird | Common Poorwill |  | 71 | 178333 | Bird | Say's Phoebe |
| 34 | 179725 | Bird | Common Raven |  | 72 | 175304 | Bird | Sharp-Shinned Hawk |
| 35 | 175309 | Bird | Cooper's Hawk |  | 73 | 177935 | Bird | Short-Eared Owl |
| 36 | 179165 | Bird | Dickcissel |  | 74 | 179532 | Bird | Snow Bunting |
| 37 | 175377 | Bird | Ferruginous Hawk |  | 75 | 177925 | Bird | Spotted Owl |
| 38 | 175407 | Bird | Golden Eagle |  | 76 | 179888 | Bird | Summer Tanager |

| **N** | **IT IS Code** | **Taxonomic Group** | **Common Name** |  | **N** | **IT IS Code** | **Taxonomic Group** | **Common Name** |
| --- | --- | --- | --- | --- | --- | --- | --- | --- |
| 77 | 175367 | Bird | Swainson's Hawk |  | 115 | 180310 | Mammal | Montane Vole |
| 78 | 179788 | Bird | Swainson's Thrush |  | 116 | 552479 | Mammal | Mountain Lion |
| 79 | 178251 | Bird | Three-Toed Woodpecker |  | 117 | 180698 | Mammal | Mule Deer |
| 80 | 175265 | Bird | Turkey Vulture |  | 118 | 180318 | Mammal | Muskrat |
| 81 | 179796 | Bird | Veery Catharus |  | 119 | 180382 | Mammal | Northern Grasshopper Mouse |
| 82 | 555388 | Bird | Western Screech-Owl |  | 120 | 179933 | Mammal | Northern Water Shrew |
| 83 | 178014 | Bird | White-Throated Swift |  | 121 | 180006 | Mammal | Pallid Bat |
| 84 | 178341 | Bird | Willow Flycatcher |  | 122 | 179954 | Mammal | Preble's Shrew |
| 85 | 176736 | Bird | Wilson's Phalarope |  | 123 | 180717 | Mammal | Pronghorn |
| 86 | 178878 | Bird | Yellow Warbler |  | 124 | 180717 | Mammal | Pronghorn |
| 87 | 177831 | Bird | Yellow-Billed Cuckoo |  | 125 | 180549 | Mammal | River Otter |
| 88 | 178964 | Bird | Yellow-Breasted Chat |  | 126 | 552496 | Mammal | Rock Mouse |
| 89 | 180109 | Mammal | American Pika |  | 127 | 180262 | Mammal | Silky Pocket Mouse |
| 90 | -3 | Mammal | Arizona Myotis |  | 128 | 180014 | Mammal | Silver-Haired Bat |
| 91 | 180008 | Mammal | Big Brown Bat |  | 129 | 179999 | Mammal | Small-Footed Myotis |
| 92 | 180086 | Mammal | Big Free-Tailed Bat |  | 130 | 180376 | Mammal | Southern Plains Woodrat |
| 93 | 180711 | Mammal | Bighorn Sheep |  | 131 | 180010 | Mammal | Spotted Bat |
| 94 | 180115 | Mammal | Black-Tailed Jack Rabbit |  | 132 | 203452 | Mammal | Townsend's Big-Eared Bat |
| 95 | 180186 | Mammal | Black-Tailed Prairie Dog |  | 133 | 180343 | Mammal | Western Harvest Mouse |
| 96 | 180582 | Mammal | Bobcat |  | 134 | 180024 | Mammal | Western Pipistrelle |
| 97 | 180222 | Mammal | Botta'S Pocket Gopher |  | 135 | 180181 | Mammal | White-Tailed Antelope Squirrel |
| 98 | 180088 | Mammal | Brazilian Free-Tailed Bat |  | 136 | 180370 | Mammal | White-Throated Woodrat |
| 99 | 179991 | Mammal | California Myotis |  | 137 | 180004 | Mammal | Yuma Myotis |
| 100 | 180201 | Mammal | Colorado Chipmunk |  |  |  |  |  |
| 101 | 180599 | Mammal | Coyote |  |  |  |  |  |
| 102 | 180122 | Mammal | Desert Cottontail |  |  |  |  |  |
| 103 | 179973 | Mammal | Desert Shrew |  |  |  |  |  |
| 104 | 179951 | Mammal | Dwarf Shrew |  |  |  |  |  |
| 105 | 180002 | Mammal | Fringed Myotis |  |  |  |  |  |
| 106 | 180184 | Mammal | Gunnison's Prairie Dog |  |  |  |  |  |
| 107 | 180017 | Mammal | Hoary Bat |  |  |  |  |  |
| 108 | 180195 | Mammal | Least Chipmunk |  |  |  |  |  |
| 109 | 179988 | Mammal | Little Brown Bat |  |  |  |  |  |
| 110 | 179990 | Mammal | Long-Legged Myotis |  |  |  |  |  |
| 111 | 180585 | Mammal | Lynx |  |  |  |  |  |
| 112 | 180559 | Mammal | Marten |  |  |  |  |  |
| 113 | 180386 | Mammal | Meadow Jumping Mouse |  |  |  |  |  |
| 114 | 180553 | Mammal | Mink |  |  |  |  |  |
